# 86. Making the APPropriate Choice: Utilization of a Smartphone Application to Optimize Antimicrobial Decisions Among Internal Medicine Trainees

**DOI:** 10.1093/ofid/ofab466.086

**Published:** 2021-12-04

**Authors:** Thomas Brooke, Herman Pfaeffle, Walter Guillory, Sorana raiciulescu, Roseanne Ressner

**Affiliations:** 1 Walter Reed National Military Medical Center, Washington, District of Columbia; 2 Uniformed Services University Biostatistics Consulting Center, Bethesda, Maryland; 3 Walter Reed National Military Medical Center, Bethesda, MD, Bethesda, Maryland

## Abstract

**Background:**

Use of an application (App) to shape antimicrobial stewardship (AS) practice is largely unknown. Walter Reed National Military Medical Center (WRNNMC) is a tertiary military academic medical center where 2020 AS guidelines transitioned to a mobile App platform. This project aimed to determine barriers to AS and the impact of an App combined with educational sessions (ES) on Internal Medicine (IM) trainee prescribing practices for common Infectious Diseases (ID) syndromes.

**Methods:**

After an orientation, participants completed a pre-intervention survey. Once weekly ES reinforcing App content was implemented over 12 weeks after which a post-intervention survey was completed. Each weekly session covered a specific ID syndrome. Survey data was analyzed using SPSS Version 27 with paired t-test.

**Results:**

Amongst 81 IM trainees, 59 (73%) completed both pre- and post-intervention surveys, of whom 39% were PGY1, 31% PGY2, and 27% PGY3. Common AS barriers included lack of knowledge, deference to seniority, established habits, and time needed to make an informed decision. The App and ES improved performance of an antimicrobial timeout (78%), IV to PO switch (61%), therapy de-escalation (56%), and antibiogram knowledge (68%) with 90% of trainees reporting increased access. Weekly ES led to 75% reporting it had at least a moderate impact on learning. Across all ID syndromes, each PGY year reported increased confidence in management post-intervention (P< 0.001) but PGY1s in particular saw the largest gain in confidence with antibiogram, febrile neutropenia, and hospital/ventilator acquired pneumonia categories. Usage of the App increased from 42% to 90% after the intervention, and 95% modified their prescribing practice based on the App. The most common barrier to App usage was forgetting to use the App.

**Conclusion:**

Utilization of an App combined with ES improved multiple domains of AS practice among IM trainees leading to a modification in antimicrobial prescribing practice in the vast majority of participants. PGY1 trainees in particular may see a large benefit which supports implementation of AS training early in the academic year. This model can be used to build a sustainable AS trainee curriculum augmenting the learning and management of common ID syndromes.

**Disclosures:**

**All Authors**: No reported disclosures

